# Depression in Chronic Suppurative Otitis Media: A Complication Brewing Unnoticed

**DOI:** 10.7759/cureus.22718

**Published:** 2022-02-28

**Authors:** Sathish Kumar, Prakash Mathiyalagen, Mathan Kaliaperumal, Sophia Mary Amalanathan, Kumaran Ramesh Colbert

**Affiliations:** 1 Department of Otorhinolaryngology, Indira Gandhi Medical College & Research Institute, Pondicherry, IND; 2 Department of Community Medicine, Indira Gandhi Medical College & Research Institute, Pondicherry, IND; 3 Psychiatry, Sri Venkateshwara Medical College and Research Centre, Pondicherry, IND

**Keywords:** chronic infection, deafness, depression, hearing loss, csom

## Abstract

Introduction: Chronic suppurative otitis media (CSOM) is a chronic infection of the middle ear cleft with a permanent perforation resulting in mucopurulent discharge and may include otalgia or fever, vertigo, hearing loss, and tinnitus. These compromise the quality of life of CSOM patients and affect their social communication and professional life also, which may induce social withdrawal and depression.

Materials & methods: This cross-sectional study was conducted at the Department of ENT, Indira Gandhi Medical College & Research Institute (IGMC&RI), Pondicherry, India, from May 2019 to May 2020. CSOM patients fulfilling the inclusion and exclusion criteria were recruited for the study.The association of disease characteristics and hearing loss was evaluated by the Spearman correlation test. Correlation of variables such as age, sex, type of disease, laterality hearing loss type, and severity with respect to depression was done using Chi-square test.

Results: One hundred forty-two participants were recruited for this study, the majority being females (65%) the mean age being 44.08 years ± SD 15. The safe type of CSOM (tubotympanic disease) was common among the participants (96%) with unilateral disease (78%) more predominating and left side being most frequently (58%). There was a significant and linear correlation of depression and hearing loss to the duration of the disease.

## Introduction

Ears are considered supreme to all other sensory organs as rightly said in couplet 411 of Thirukural: “Wealth of wealth is wealth acquired be ear attent; wealth amid all wealth supremely excellent," the importance of which is not realized until lost. Many diseases and disorders affect the ear and subsequently hearing. Chronic suppurative otitis media (CSOM) contributes a major share to this burden globally both in underdeveloped and developed countries. It is defined as a chronic infection of the middle ear cleft with a permanent perforation resulting in mucopurulent discharge [1]. The incidence of CSOM is 4.76%, which equates to 31 million cases per year. A World Health Organization (WHO) report suggests that 65 to 350 million individuals suffer from CSOM globally, and the disease is a leading cause of hospital visits [2].

CSOM presents with a chronically discharging ear for at least four to six weeks, with an antecedent history of recurrent acute otitis media (AOM), traumatic perforation, or insertion of grommets [3]. Malodorous ear discharge may induce social withdrawal and depression [4]. Other symptoms are otalgia, fever, vertigo, hearing loss, and tinnitus. All of these symptoms compromise the quality of life in individuals with CSOM and also affect their social communication and professional life. The effect of CSOM on hearing is well known. Many studies have focused on the association of depression with hearing loss and its effect on health-related quality of living. CSOM being the leading cause of hearing loss across the world and the paucity of literature on the association of CSOM to depression directed us to investigate the prevalence of depression, grade its severity in CSOM patients and establish a cause-effect relationship. Other risk factors that could contribute to depression were socioeconomic status, duration of disease, and occupational stress [5,6].

## Materials and methods

This study is a cross-sectional analytical study. It was done on subjects attending the out-patient department of Otorhinolaryngolgy, at Indira Gandhi Medical College & Research Institute (IGMC&RI), for the treatment of CSOM, from May 2019 to May 2020. Our institute is a tertiary care hospital in the South Indian city of Pondicherry, catering to the needs of the local residents and also from the neighbouring districts of Tamilnadu State. All subjects in the age group of 20 to 50 years diagnosed with CSOM in at least one ear were included in the study. Subjects under the age of 20 and above 50 years were excluded from the study in order to have an otherwise healthy and mature study group. We also excluded subjects with previous history or family history of psychological disorder or on treatment for psychological disorders and those with comorbidities such as diabetes, hypertension, heart disease. Since the study was planned with a questionnaire to be filled by the subjects themselves under supervision of a clinician, we excluded those with educational qualification of <8th standard.

Assuming the proportion of CSOM patients having depression as 50% with alpha error as 5%, an error of margin as 9%, and a non-response rate of 10% the minimum sample size was calculated to be 131 based on the formula n=Z2P(1-P)/d2, Where n is the sample size, Z is the statistic corresponding to the level of confidence, P is expected prevalence, and d is precision. The actual sample we achieved was 142. Subjects diagnosed with CSOM fulfilling the inclusion criteria were selected. A detailed history was taken regarding the onset, duration, progression, nature of treatment received so far, and also complications of CSOM if present. The subjects were then assessed clinically for laterality (unilateral or bilateral) of disease, type of CSOM, and stage of the disease at presentation. Otoscopy was performed to document the tympanic membrane findings. The subjects then underwent pure tone audiometry to assess the type and degree of hearing loss, the findings of which, were documented for analysis. The subjects were then given a pre-validated structured questionnaire, the Hamilton Depression Rating Scale (HDRS), to be filled under the supervision of a clinician to assess depression. The scores obtained were documented in the proforma. The data was analysed using Statistical Package for Social Sciences (SPSS) version 20 (IBM Corp., Armonk, NY, USA). The association between duration disease, type of disease, CSOM related complications, and hearing loss was evaluated by the Spearman correlation test. A Chi-square test was done to assess significant differences among variables such as age, sex, type of disease, laterality, hearing loss type, and severity concerning depression.

## Results

One hundred forty-two participants were recruited for this study, the majority being females (65%) and residents of Pondicherry state (81%); the mean age was 44.08 years ± SD 15. The safe type of CSOM (tubotympanic disease) was predominantly observed among the participants (96%) with unilateral disease (78%) more common than bilateral (22%) and the left side was found to be most frequently affected (58%) (Table 1).

**Table 1:** Demographic and disease characteristics with and without depression CSOM: chronic suppurative otitis media

VARIABLE	DEPRESSION		TOTAL	CHI SQUARE TEST
	YES	NO		
SEX				
FEMALE	28 (65%)	64 (65%)	92 (65%)	P = 0.957
MALE	15 (35%)	35 (35%)	50 (35%)	
RESIDENCE				
PONDICHERRY	32 (74%)	83 (84%)	115 (81%)	P = 0.189
TAMILNADU	11 (26%)	16 (16%)	27 (19%)	
CSOM TYPE				
SAFE	43 (100%)	94 (95%)	137 (96%)	P = 0.323
UNSAFE	0	5 (5%)	5 (4%)	
LATERALITY				
BILATERAL	14 (33%)	17 (17%)	31 (22%)	P =0.108
LEFT	18 (42%)	46 (46%)	64 (45%)	
RIGHT	11 (25%)	36 (37%)	47 (33%)	
PREVIOUS EAR SURGERIES				
NO	41 (95%)	94 (95%)	135 (95%)	P = 1
YES	2 (5%)	5 (5%)	7 (5%)	
COMPLICAITONS OF CSOM				
NO COMPLICATIONS	43 (100%)	97 (98%)	140 (98%)	P = 0.644
FACIAL PALSY	0	1 (1%)	1 (1%)	
MASTOIDITIS	0	1 (1%)	1 (1%)	

Seven subjects (5%), a small proportion of the study population, had undergone ear surgeries in the past for CSOM and only two (1%) were cured of the disease, the rest had a recurrence. CSOM related complications were observed in two participants (1%), which were lower motor neuron type of facial palsy and mastoiditis respectively (Table 1). More than one-fourth of participants (27%) had suffered from the disease for more than 15 years although a majority (54%) had the disease for less than a year (Table 2). On audiological evaluation mild to moderate hearing loss was the most frequent grade of hearing loss observed and a small proportion (4%) had severe to profound hearing loss (Table 3).

**Table 2:** Correlation of laterality and duration of disease with depression

VARIABLE	DEPRESSION		TOTAL	CHI SQUARE TEST
	YES	NO		
LATERALITY				
BILATERAL	14 (32%)	17 (17%)	31 (22%)	P = 0.041
UNILATERAL	29 (68%)	82 (83%)	111 (78%)	
DURATION				
1-5 YR	11 (25%)	32 (32%)	43 (30%)	P = 0.039
10-15 YR	2 (5%)	3 (3%)	5 (3%)	
5-10 YR	6 (15%)	10 (10%)	16 (11%)	
LESS THAN 1 YR	11 (25%)	43 (44%)	54 (39%)	
MORE THAN 15 YR	13 (30%)	11 (11%)	24 (17%)	

**Table 3:** Correlation of degree of hearing loss with depression

VARIABLE	DEPRESSION		DEPRESSION	
	YES	NO	YES	NO
HEARING LOSS	RIGHT EAR		LEFT EAR	
MILD	6 (15%)	28 (29%)	10 (23%)	29 (29%)
MODERATE	9 (21%)	22 (22%)	14 (33%)	20 (20%)
MODERATELY SEVERE	11 (25%)	19 19%)	9 (21%)	21 (21%)
NORMAL	13 (30%)	30 (30%)	7 (17%)	26 (27%)
PROFOUND	1 (2%)	0	1 (2%)	0
SEVERE	3 (7%)	0	2 (4%)	3 (3%)
CHI SQUARE TEST	P = 0.030		P = 0.295	

We observed a linear correlation of depression and hearing loss to the duration of the disease. As the duration of the disease increased there was a significant adverse progression of hearing impairment and also the severity of depression. We also observed that as hearing impairment increased there was a significant progression of depression (Table 4).

**Table 4:** Correlation of depression score with duration of disease for linear trend

		TOTAL DURATION					Total
		LESS THAN 1 YR	1-5 YR	5-10 YR	10-15 YR	MORE THAN 15 YR	
HAMILTON DEPRESSION SCORE	Normal	43	32	10	3	11	99
	Mild	8	9	3	2	10	32
	Moderate	2	2	3	0	2	9
	Severe	1	0	0	0	1	2
Total		54	43	16	5	24	142
Chi-square for linear trend; p value=0.006							

The prevalence of depression in our study was 30%, females being more commonly (65%) affected than males (Table 1). The majority (74%) had mild depression, nine (21%) had moderate and two (5%) had severe depression (Table 4). On analyzing the effect of the laterality of disease on depression, we observed a significant difference. The participants having bilateral disease were more prone to develop depression than those with unilateral disease (Table 2). Although depression was more prevalent among the female participants in comparison to their male counterparts, there was no significant correlation observed. We could not observe any significant effect of the type of disease, previous surgeries, and the presence of CSOM-related complications on depression (Table 1).

## Discussion

The prevalence of CSOM in developing countries is estimated to be 72 per 1000. The expenditure on treating ear infections is estimated to be more than 2 billion dollars in the US every year with a prevalence rate of less than 30 per 1000 [5,7].

Parikh et al. [8] in their study stated that any bodily infirmity/loss of function can lead to social withdrawal, and lack of motivation culminating in depression if left unattended. Ears are no exception to this. Loss of their function in the form of hearing loss and persistent/recurrent ear discharge in CSOM causes a similar effect and may lead to depression.

Depression is characterized by the presence of depressed mood and/or lack of interest in activities that would usually be pleasurable, with loss of appetite, sleep disorders, feeling of guilt and/or depreciation, and all these lasting for at least two weeks [8,9].

Depressive disorders are emerging as a significant public health problem not only due to their increasing prevalence but also due to the cost incurred for treating it either directly or indirectly [10]. Projections of the year 2020 indicate that major depression will occupy second place in terms of impact on human health seconding ischemic heart disease. If incapacity alone is taken into consideration, major depression topped the list in 1990 [11].

Depressive disorders can lead to a lack of work productivity and interpersonal problems are serious consequences of depressive disorders in terms of secondary morbidity [12]. There are strong evidences available in the past literature that depressive disorder leads to a reduction in quality of life (QOL) [13-15].

Numerous studies have focussed on the association of depression and hearing loss [15,16] and also the association of health-related quality of life in people suffering from CSOM [17-19]. But there were only a few studies that focussed on depression associated with the disease per se. We attempted to grade the severity of depression among CSOM patients using the Hamilton Depression Rating Scale and establish a cause-effect relationship, and we did succeed.

In CSOM, hearing loss occurs due to two phenomena; one is due to lack of effective conduction of sound waves due to tympanic membrane perforation causing conductive hearing loss, and the other is sensory end organ damage due to toxins and inflammatory mediators causing a mixed type of hearing loss [20].

The hearing loss resulting from CSOM restricts a person’s ability to communicate effectively leading to depression, anxiety, and social withdrawal [21,22]. Hearing loss along with frequent ear discharge, medical consults, expenses incurred for investigations and treatment have been found to significantly affect the health-related quality of life in various dimensions viz physical, functional, social, psychological, and familial [23,24].

It was earlier thought that the depression resulting from hearing loss is because of the physical, mental and financial handicaps. But, of late there are shreds of evidence in the literature that demonstrated that hearing loss can induce plasticity in the excitatory and inhibitory neurotransmitter systems (serotonin) in the central auditory brain regions [25,26]. The study by Ozturk et al. showed that selective serotonin reuptake inhibitors (SSRIs) like sertraline given for treating depression have a protective effect against cisplatin-induced ototoxicity. The study by Rao et al. demonstrated that acoustic trauma in an animal model induced substantial hearing loss and caused selective upregulation of serotonin receptor genes in the inferior colliculus [26]. The information on the networks and etiologic mechanisms involved is still scant and needs further research.

Mehboob et al. [27] assessed the association of depression, anxiety, and stress in patients with CSOM by comparing the treatment effect among the study groups. There was a significant correlation between hearing loss and gender, that is, female patients suffered from hearing loss more than males. Such difference was not observed in our study. The severity of hearing loss increases with the age of patients similar to that of ours.

Though the symptoms of depression were observed more among females than males similar to the earlier studies [28], it was not statistically significant in our study. Depression among these subjects was a result of frustration and hopelessness about the disease (CSOM) and lack of motivation. Since the majority of subjects had only mild depression, they did not require any intervention, but just counseling. Those subjects having moderate and severe depression were treated as advised by the psychiatrist and were followed up regularly.

One major limitation observed with the HDRS was that it did not include some of the atypical symptoms of depression. Data on other risk factors which could lead to hearing loss such as the presence of other chronic non-communicable diseases, smoking, chronic noise exposure and ototoxicity was lacking. The financial or socioeconomic status of subjects was also not taken into account. All these factors could possibly affect the subject’s quality of living either individually or together and thus, may lead to depression.

## Conclusions

We conclude that a long-standing ear disease with persistent discharge and hearing impairment can result in depression. The severity of depression increases proportionately with increasing duration of disease, severity of disease and the degree of hearing impairment. We believe this study will help to deliver better and timely care to curtail the suffering from CSOM and to identify susceptible individuals early in the course of the disease to treat them holistically. We expect that this study will provoke and promote further prospective clinical and biomedical studies on the relationship of CSOM, hearing loss, and depression to determine its aetiopathogenesis.
